# Machine Learning Models for Cancer Research: A Narrative Review of Bulk RNA-Seq Applications

**DOI:** 10.3390/ijms262412081

**Published:** 2025-12-16

**Authors:** Elena A. Pudova, Vladislav S. Pavlov, Zulfiya G. Guvatova, Maria S. Fedorova, Petr V. Shegai, Anna V. Kudryavtseva, Anastasiya V. Snezhkina

**Affiliations:** 1 Engelhardt Institute of Molecular Biology, Russian Academy of Sciences, 119991 Moscow, Russia; 2 National Medical Research Radiological Centre of the Ministry of Health of the Russian Federation, Koroleva St. 4, 249036 Obninsk, Russia; 3 Scientific and Educational Resource Center for Innovative Technologies of Immunophenotyping, Digital Spatial Profiling and Ultrastructural Analysis, Patrice Lumumba Peoples’ Friendship University of Russia (RUDN University), 117198 Moscow, Russia

**Keywords:** machine learning, deep learning, bulk RNA-Seq, expression models, cancer

## Abstract

Integrating the advantages of machine learning with the rapidly accumulating high-throughput sequencing data facilitates our capacity for biological discovery and the advancement of molecular medicine. In recent years, bulk RNA-seq technology has established itself as a cost-effective and widely used method for obtaining complete transcriptome profiles of test samples, enabling the identification of key cancer-associated expression patterns. Various machine learning algorithms, in turn, enable the development of informative diagnostic and prognostic models, ensuring the efficient processing of high-dimensional RNA-Seq data. The convergence of these methods shows great promise for oncology. In this narrative review, we describe bulk RNA-Seq-based ML models in oncology as a complete workflow from data preprocessing to model validation. We provide practical recommendations for algorithm selection and study design, and discuss bulk RNA-Seq deconvolution as a cost-effective alternative to single-cell RNA-Seq for analyzing tumor cellular composition. These insights offer a practical guide for developing reproducible diagnostic and prognostic models with translational potential.

## 1. Introduction

Cancer is a complex disease characterized by the uncontrolled growth and spread of malignant cells. In recent decades, the prevalence of the disease has increased rapidly, and it is projected that by 2050, the number of new cancer cases could reach 35 million [1]. The heterogeneity of various types of cancers is evident at both the clinical and molecular levels. This complicates the process of diagnosis and disease prognosis, necessitating the application of integrative molecular methods capable of accounting for the multiple characteristics of the disease [2]. Bulk RNA sequencing (bulk RNA-Seq) is a powerful tool for studying the molecular mechanisms of cancer development and progression. This method is employed in over 60% of next-generation sequencing-related research projects and provides detailed mapping of the transcriptomic landscape, identification of gene expression changes, and analysis of molecular pathways involved in the development of various diseases, including cancer [3].

RNA-Seq offers several advantages over other methods for the assessment of gene expression. Firstly, it allows for the simultaneous analysis of various RNA fractions, including coding transcripts, non-coding regulatory RNAs, and functional RNAs. The high resolution and sensitivity enable the detection of even minor changes in gene expression, while its wide dynamic range ensures accurate quantification of transcription levels even for rare transcripts [4]. To date, various types of RNA-Seq approaches have been developed, such as single-cell RNA-Seq (scRNA-Seq), laser capture micro-dissected RNA-Seq (LCM RNA-Seq), digital spatial profiling technology (Stereo-Seq), and others [5,6,7]. Nevertheless, bulk RNA-Seq remains the most cost-effective and widely utilized tool in both fundamental cancer research and clinical applications [3].

With the development of sequencing technologies, numerous public multi-omics databases have been created, combining transcriptomic, genomic, and epigenomic data. The Cancer Genome Atlas (TCGA) database is of particular significance within this field, as it contains molecular and clinical data from over 11,000 patients with 33 different cancer types [8]. Similarly, the Gene Expression Omnibus (GEO) is a repository that includes microarray, NGS, and other high-throughput functional genomics data on various disorders [9]. The availability of these data has been demonstrated to significantly accelerate the development of new diagnostic tools and targeted therapies, thereby opening up new research opportunities. Nevertheless, despite the substantial accumulation of diverse transcriptomic data, their analysis remains limited. In order to address the challenges associated with the processing of large datasets, the identification of hidden patterns, and the integration of heterogeneous molecular profiles, there is a necessity for more advanced analytical approaches.

Machine learning (ML) is a field of artificial intelligence concerned with the identification of patterns in data and the improvement of algorithms based on acquired knowledge [10]. ML has emerged as a potent instrument for the automation of RNA-Seq data processing, enhancing the precision of detecting biologically significant gene expression patterns and optimizing the interpretation of results in biomedical research [11,12]. The application of ML in RNA-Seq data analysis has already significantly advanced the search for new informative diagnostic and prognostic markers and the development of personalized therapeutic strategies [13,14]. Nevertheless, despite the growing number of studies in this field, several challenges remain to be addressed. The challenges encountered primarily pertain to data preprocessing, the selection of optimal algorithms, and the interpretability of the results.

In this narrative review, we focus on ML models developed using bulk RNA-Seq data in oncology. The main aim of the work is to present existing studies as a coherent methodological pipeline—from data preprocessing through feature selection, algorithm choice, model training, and validation. We place particular emphasis on situations in which ML and deep learning methods genuinely offer advantages over traditional statistical approaches. We also highlight critical nuances of the ML workflow for bulk RNA-Seq data and their impact on the reproducibility and clinical applicability of models. A separate focus is placed on bulk RNA-Seq deconvolution methods as a practical alternative to scRNA-Seq for characterizing the cellular composition of tumors when access to single-cell technologies is limited. Based on this analysis, we formulate practical recommendations for algorithm selection and preprocessing design in oncology studies, and we outline key limitations and future directions for the development of bulk RNA-Seq—based models. The present work can facilitate a better understanding of the capabilities and limitations of current diagnostic and prognostic tools based on transcriptomic data and will be of particular interest to both research and clinical oncology communities.

### Review Methodology

This review summarizes studies on RNA-Seq data-based model development in oncology using various ML algorithms published between 2019 and 2025. A literature search was conducted using the PubMed database of the US National Library of Medicine (https://pubmed.ncbi.nlm.nih.gov, accessed on 1 September 2025) to identify articles published between 1 January 2019, and 1 January 2025, using a combination of the keywords “RNA-seq”, “machine learning”, “deep learning”, “cancer”, “models” and “oncology”. Initially, more than 100 articles were screened by titles and abstracts according to the specified criteria, from which approximately 40 key studies were selected, primarily on the direct use of bulk RNA-Seq data for model development. Our research analysis aims to provide a narrative synthesis of trends in RNA-Seq data preprocessing, the use of the most significant traditional ML algorithms, and specialized deep learning models in oncology research without conducting a quantitative meta-analysis. The primary goal of our design for this review is to provide a comprehensive overview of the application of bulk RNA-Seq data for researchers and clinicians beginning to explore this field.

## 2. Preprocessing of RNA-Seq Data

RNA-Seq is a technique that generates high-dimensional data, with measurements pertaining to the expression of thousands of transcripts across a range of samples. The data are characterized by high complexity and redundancy, as the number of transcripts significantly exceeds the number of samples. On the one hand, the comprehensiveness of RNA-Seq data enables a wide range of applications for diverse research questions using various bioinformatic tools. Conversely, the intricate nature of RNA-Seq data poses significant challenges in the development of ML models, including the increased risk of overfitting (where a model adapts too closely to the training data, loses its ability to generalize, and performs poorly on new data).

**Figure 1:**
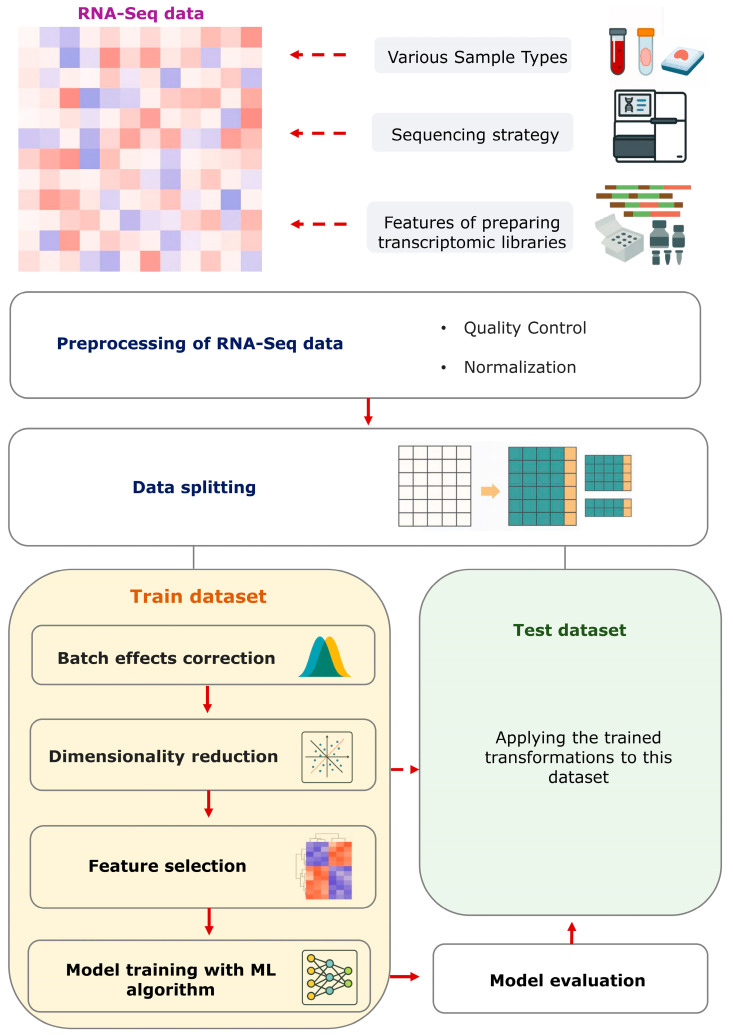
Schematic pipeline for RNA-Seq data preprocessing and ML model development. Bulk RNA-Seq data are characterized by high heterogeneity depending on various technical and biological factors. At the first stage, basic preprocessing is required, including quality control and data normalization. After these steps, the data are split into a training set and an independent test set. All subsequent steps, including batch-effect correction, dimensionality reduction and feature selection for the model, are optimized exclusively on the training set and then applied unchanged to the test set. This ordering preserves the independence of the test set, prevents data leakage and provides a reproducible basis for developing informative models using bulk RNA-Seq data.

Preprocessing constitutes a pivotal component in the adaptation of RNA-Seq data for subsequent analysis, with the objective of enhancing data quality, reducing noise, and augmenting the robustness of the developed models [15] (Figure 1). Moreover, the intrinsic quality of RNA-Seq data is itself a prerequisite for reliability, which primarily depends on the quality of the original RNA samples, the choice of the type of transcriptome library preparation (poly(A) selection or rRNA depletion), and their correct preparation from the biological samples under study [16].

Another critical factor determining the accuracy of transcript quantification is sequencing depth. Equally important is the design of the sequencing experiment, which includes the selection of an optimal strategy based on the analytical objectives. Careful planning of sequencing experiments is essential to avoid technical errors, especially in studies involving a significant number of samples processed over multiple sequencing runs [16].

Normalization of RNA-Seq data is crucial to eliminate technical and biological variations. To compensate for differences in sequencing depth between samples, various approaches are used, such as RPKM (Reads Per Kilobase of exon model per Million mapped reads), FPKM (Fragments Per Kilobase of exon model per Million mapped reads), and TPM (Transcripts Per Million). However, the effectiveness of these methods decreases when transcripts are unevenly distributed across samples. This is due to the fact that transcripts with high levels of expression can skew the distribution [16,17]. It is evident that contemporary normalization methodologies, including TMM (Trimmed Mean of M-values), DESeq, PoissonSeq and UpperQuartile, are capable of accounting for the distinctive characteristics inherent in RNA-Seq data [16,18,19]. The transcripts exhibiting elevated variability and/or high expression are disregarded, thereby enhancing the robustness of the system to such biases. Despite the process of normalization, batch effects resulting from technical variations between experimental batches may persist. These effects have the potential to obscure genuine biological variations, thereby compromising the accuracy of data analysis. It has been demonstrated that methods such as COMBAT and ARSyN correct for these variations, thereby enhancing data comparability across different experimental batches and improving the reproducibility of results [20,21].

The high dimensionality of RNA-Seq data presents a significant challenge to their direct application in ML algorithms. Dimensionality reduction is a pivotal step in the processing of RNA-Seq data, whereby it is transformed into a space with a reduced number of dimensions. This process retains essential information while eliminating noise and redundant features. The reduction in dimensionality can be categorized into two distinct methods: linear and nonlinear. Linear approaches include principal component analysis (PCA) and its modifications such as sparse PCA, as well as independent component analysis (ICA) and non-negative matrix factorization (NMF) [22,23,24,25]. PCA makes it possible to identify components that explain the largest proportion of variance and is widely used to visualize sample structure, detect outliers, and reveal major sources of variation. In turn, sparse PCA extends this idea by imposing penalties on loadings, yielding components based on a relatively small set of genes and thereby simultaneously performing coarse feature selection. ICA decomposes the expression matrix into statistically independent components and is useful for identifying modules of co-regulated genes, as well as for separating technical and biological sources of variation. Unlike PCA and ICA, NMF imposes non-negativity constraints on the factors and provides an additive decomposition of the expression matrix, allowing the resulting components to be interpreted as «metagenes». Nonlinear methods have been developed to address this limitation, with the capacity to capture complex nonlinear patterns. Among the most common nonlinear methods are t-SNE and UMAP, which facilitate the visualization of complex cluster structures based on RNA-Seq data [26,27]. The selection of a suitable tool is contingent upon the nature of the dataset in question.

Despite the fact that RNA-Seq data contain information on the expression of thousands of transcripts, only a subset of these contributes meaningful biological insight for a given research task. The performance of models constructed directly using conventional ML algorithms is contingent on the preliminary feature selection step, which involves the identification of a group of transcripts whose expression most accurately represents the dataset’s structure in a lower-dimensional space and enhances the signal-to-noise ratio [28].

Filter methods signify the primary and most elementary approach to feature selection, whereby each feature is evaluated independently based on statistical criteria. A plethora of statistical methods, including variance analysis, the Mann–Whitney U test, *t*-tests, the Kruskal–Wallis test, and correlation coefficients, are employed for the preliminary exclusion of uninformative transcripts [29,30,31]. However, it should be noted that filter methods have been shown to be suboptimal in a number of cases, as they have been observed to ignore complex interdependencies between features within the RNA-Seq data structure [32]. This has the potential to result in the loss of biologically significant information. The utilization of wrapper methods has been demonstrated to circumvent this limitation by evaluating feature subsets within the context of a designated classification algorithm. Recursive feature elimination, sequential feature selection, and genetic algorithms iteratively optimize the feature set, maximising model performance [32]. However, the application of these methods is constrained by two main factors. Firstly, they are characterized by high computational complexity. Secondly, there is a risk of overfitting, which is especially relevant when the sample size is small. Embedded methods combine the strengths of filter and wrapper approaches by integrating the model training process with the feature selection procedure, enabling the effective identification of optimal feature subsets while accounting for their interactions [33]. One of the most widely utilized embedded methodologies for RNA-Seq data analysis is the Least Absolute Shrinkage and Selection Operator (LASSO). The proposed methodology involves the incorporation of a penalty (L1-regularization) within the regression model. This penalty serves to force the coefficients of non-informative features to zero, consequently excluding them from the analysis. This feature reduction has been demonstrated to lower the risk of overfitting, thus rendering LASSO particularly effective for high-dimensional RNA-Seq data [34,35,36,37]. However, the propensity of LASSO to randomly select a single feature from a group of correlated genes prompted the development of the Elastic Net algorithm, which integrates L1 and L2 regularization to facilitate more stable selection of groups of interrelated features [38].

## 3. Conventional ML Algorithms in Cancer Studies

Despite the growing interest in deep learning (DL) models, the use of conventional ML algorithms continues to be actively applied for solving various tasks in oncology research, particularly for the analysis of RNA-Seq data. The prevalence of these algorithms can be attributed to two key factors: the relative simplicity of interpreting their results and the relatively modest requirements for the size of the training sample.

A recent analysis of relevant studies has revealed a clear trend towards the utilization of hybrid feature selection strategies (Table 1). The majority of studies employ the LASSO regression algorithm in combination with other methods, such as initial filtering through differential gene expression analysis or the construction of protein–protein interaction networks, to identify functionally related modules [39,40]. Such multi-stage strategies enable researchers to achieve effective dimensionality reduction of RNA-Seq data down to compact gene panels. It is important to note that the size of a gene panel does not always directly correlate with model accuracy. It has been demonstrated that certain models incorporating a limited number of genes achieve an Area Under the Curve (AUC) above 0.95, while others may require dozens of markers to reach equivalent performance levels [39,40].

Among the most frequently used algorithms for model construction, Random Forest (RF) remains prominent, as evidenced by its presence in the majority of the analyzed studies. The extensive utilization of this ensemble algorithm can be ascribed to its substantial resilience to overfitting and its notable accuracy, particularly in the context of binary classification tasks. Moreover, the interpretability of the algorithm is enhanced by its integrated feature importance assessment, a feature that is critical for the translation of results into clinical practice, where comprehension of the biological basis of a decision is frequently more significant than absolute accuracy [13,40,41]. The Support Vector Machine (SVM) algorithm is the second most popular and has been shown to be particularly effective in tasks with limited sample sizes. The integration of the SVM algorithm with multi-stage feature selection, employing methodologies such as mutual information and recursive feature elimination, has been demonstrated to yield enhanced outcomes. For instance, an accuracy of 97.99% with an AUC of 0.99 was achieved for an expression model diagnosing lung adenocarcinoma [42].

**Table 1:** RNA-Seq-based models developed using conventional ML algorithms in the field of oncology encompass the domains of diagnosis, prognosis and classification.

Cancer Type	Model Type	Datasets and Cohort Size	Sample Type	Feature Selection Methods	Model Algorithm	Metrics	Signature Size	External Validation	Ref.
Colorectal cancer	Diagnostic	TCGA-CRC (*n* = 695), GSE50760 (*n* = 54), external GSE142279 (*n* = 40)	Tumor and normal tissues	LASSO, gene expression, correlation and survival analyses	Adaboost, RF, LR, GNB, SVM (final RF)	Internal (TCGA-CRC): RF accuracy 100%, internal (GSE50760): RF accuracy 94.44%, external (GSE142279, 3-gene RF model): accuracy 100%, gene AUC 0.99–1.00	3 genes	Yes (GSE142279, RF classifier)	[40]
Colorectal cancer	Diagnostic	GSE10950, GSE25070, GSE41328, GSE74602, GSE142279—train (*n* = 154), validation (*n* = 66), external GSE21815 (*n* = 141), GSE106582 (*n* = 194)	Tumor and normal tissues	DEG, PPI (STRING), LASSO	RF, SVM, ANN, GBM	Internal (balanced train/validation): AUROC > 0.95 for RF, SVM, ANN, GBM, external GSE21815 (SVM): AUROC 0.982, accuracy 0.978, external GSE106582 (RF): AUROC 0.980, accuracy 0.958	9 genes	Yes (independent cohorts GSE21815, GSE106582)	[39]
Colorectal cancer	Diagnostic	GSE44861 (*n* = 111), GSE103512 (*n* = 69), TCGA-COAD/READ (*n* = 698)	Tumor and normal tissues	MCFS, Boruta, mRMR, LightGBM	SVM, XGBoost, RF, kNN, interpretable rules	10-fold CV: accuracy 81.2–98.6%, AUC 0.82–1.00 (best models with AUC ≈ 0.99)	33, 52, 49 genes	No	[43]
Lung adenocarcinoma	Diagnostic and prognostic	TCGA-LUAD (*n* ≈ 500), external GSE7670 (*n* = 54), GSE30219 (*n* ≈ 300), GSE31210 (*n* = 226), GSE50081 (*n* = 127), GSE37745 (*n* = 106)	Tumor and normal tissues	LASSO, RF, SVM	KNN, NB, RF, DT, SVM, XGBoost (diagnostic); LASSO–Cox (prognostic)	Diagnostic models: 10-fold CV on training set AUC-ROC 0.95–1.00, AUC-PRC 0.98–1.00, accuracy > 95%; test set AUC-ROC 0.93–1.00, AUC-PRC 0.99–1.00, accuracy > 95%. Prognostic model: time-dependent ROC AUC ~0.78 at 1 year and ~0.73 at 2–4 years	13 markers in the diagnostic model, 12 genes in the prognostic model	Yes (GSE7670 for diagnostic model; GSE30219, GSE31210, GSE50081, GSE37745 for prognostic model)	[41]
Lung adenocarcinoma	Diagnostic	TCGA-LUAD (*n* = 549), external GSE81089 (*n* = 54)	Tumor and normal tissues	MI, RFE, RF	SVM, RF	Internal (TCGA test set, 12-gene model): accuracy ≈ 97.99%, balanced accuracy ≈ 90.66%, AUC ≈ 0.993, external (GSE81089): accuracy ≈ 96.29%, balanced accuracy ≈ 94.44%, AUC = 1.00	12 genes	Yes (GSE81089)	[42]
Multiple tumor types	Classification	Pan-paediatric RNA-seq cohorts covering 52 tumour types and 96 tumour subtypes, external KidsFirst paediatric tumour dataset	Tumor tissues	ANOVA (F-statistic), variance, PCA, RF feature importance	Weighted RF, weighted kNN, ensemble	Overall tumour-type classification: internal accuracy 94–99%, precision~99%, recall~80%, subtype classification: accuracy ≈ 86%. External KidsFirst cohort: precision 98%, recall 77%	300 transcripts + PCA components	Yes (KidsFirst)	[44]
Multiple tumor types	Classification	UCI ML Repository (RNA-Seq HiSeq PANCAN, *n* = 801)	Tumor tissues	ANOVA F-test, PCA	Decision Trees, SVM, LR, Naive Bayes, KNN, ensembles, wide neural networks	Internal train/validation/test splits: best wide neural network—accuracy 99.834% (validation) and 99.995% (test), several models reach accuracy up to 100% with AUC up to 1.00 (five-class tumour-type classification)	16,345 features after selection + PCA (95% explained variance)	No	[45]
Breast cancer	Classification, Prognosis	TCGA-BRCA (*n* = 376), CCLE (*n* = 80)	Tumor tissues and cell lines	Alternative splicing (PSI of cassette exons), differential splicing, Boruta (RF importance)	Semi-supervised RF (cell-to-patient transfer learning), univariate Cox regression	Cell-to-patient RF classifier using a 25-event splicing signature stratified basal-like patients: 5-year DSS HR = 4.87 (95% CI 1.37–17.28), log-rank *p* = 0.0067	25 splicing events	No	[46]
Lung squamous cell carcinoma	Prognostic and predictive	TCGA-LUSC (*n* = 478), external GSE30219, GSE37745, GSE73403 (combined *n* = 200)	Tumor tissues	LASSO, RF	Multivariate LR	TCGA training set: AUC = 0.967, accuracy 89.9%, TCGA testing set: AUC = 0.956, accuracy 88.9%, external GEO cohorts: classifier separates two stemness subtypes with clearly different OS	9 genes	Yes (GSE30219, GSE37745, GSE73403)	[47]
Pancreatic cancer	Prognostic	TCGA-PAAD (*n* = 182), GTEx-PAAD (*n* = 167), external GSE62452 (*n* = 130), GSE28735 (*n* = 90)	Tumor and normal tissues	Univariate Cox, LASSO, Gaussian finite mixture model	Multivariate Cox regression, Kaplan–Meier	TCGA-PAAD: 5-gene risk score AUC ≈ 0.75 for OS, external GSE62452 + GSE28735: AUC ≈ 0.91	5 genes	Yes (GSE62452, GSE28735)	[48]
B-cell lymphoma	Prognostic	GSE10846 (*n* = 233), GSE23501 (*n* = 64)	Tumor tissues	Univariate Cox, Mclust, RF	RF survival model	C-index = 0.84 (training), 0.79 (validation)	54 genes	Yes (GSE23501)	[49]
Prostate cancer	Prognostic	RNA-Seq LAPC cohort from Russian patients (*n* = 73), independent FFPE LAPC cohort (*n* = 37)	Tumor tissues (fresh frozen and FFPE)	DEGs, statistical tests	LR, LGBM, CatBoost, RF, XGBoost	Best model “CST2 + OCLN + pT” (CatBoost): internal AUC ≈ 1, external FFPE cohort: AUC = 0.863, accuracy 0.81, sensitivity 0.83, specificity 0.79	2 genes + clinical feature	Yes (independent FFPE cohort)	[13]
Lung adenocarcinoma	Prognostic	TCGA-LUAD (*n* = 547), external GSE31210, GSE30219, GSE50081 (total *n* = 369)	Tumor tissues	Univariate Cox, correlation analysis, Lasso, RSF, Elastic Net	Elastic Net Cox model (27-lncRNA risk score)	TCGA-LUAD: C-index = 0.677 (95% CI 0.63–0.73), time-dependent AUC = 0.76 (1 year), 0.72 (2 years), 0.74 (3 years), external GSE31210, GSE30219, GSE50081: C-index > 0.67 and significant OS/DFS separation between risk groups	27 lncRNAs	Yes (GSE31210, GSE30219, GSE50081)	[50]
Clear cell renal cell carcinoma	Prognostic	TCGA-KIRC (*n* = 614)	Tumor tissues	Univariate Cox, LASSO, multivariate Cox	LASSO-Cox	TCGA-KIRC: C-index = 0.783 (95% CI 0.775–0.816), time-dependent AUC = 0.725 (1 year), 0.718 (3 years), 0.762 (5 years)	4 lncRNAs	No	[51]
Breast cancer	Prognostic	TCGA-BRCA (1113 cases), external GSE20685 (*n* = 327), IMvigor210, GSE78220	Tumor tissues	Univariate Cox, RSF, stepwise multivariate Cox	Multivariate Cox	TCGA-BRCA: time-dependent AUC for OS = 0.78 (1 year), 0.67 (3 years), 0.63 (5 years), nomogram C-index ≈ 0.79, external GSE20685: 1-/3-/5-year AUC ≈ 0.81/0.66/0.70	5 genes	Yes (GSE20685)	[52]
Multiple tumor types	Prognostic and predictive	TCGA with clinical drug response data (five cancer types), 5-FU model: train (*n* = 58), validation (*n* = 17), gemcitabine (GCB) model: train (*n* = 92), validation (*n* = 28)	Tumor tissues	Clara (OptCluster), RF	RF	Pan-cancer RF models: CV AUC = 0.98 for both 5-FU and GCB, independent validation: 5-FU model AUC 0.56, accuracy 52.9%, GCB model AUC 0.71, accuracy 85.7%	2 gene clusters	No	[53]
Gastroenteropancreatic neuroendocrine tumors	Diagnostic and prognostic	GSE98894 (*n* = 182), GSE118014 (*n* = 32)	Tumor tissues	mRMRe	RF, SVM, LDA, GBM, XGB, kNN, CART	RF-based models for hepatic metastasis and primary site: internal accuracy up to 100%, external validation (GSE118014): accuracy > 90% for metastasis classification and >95% for primary-site prediction	21 genes (9 for metastasis, 12 for primary tumor)	Yes (GSE118014)	[54]

Abbreviations: AUC, Area Under the Curve; BRCA, Breast Cancer; CART, Classification and Regression Trees; CCLE, Cancer Cell Line Encyclopedia; C-index, Concordance Index; COAD, Colon Adenocarcinoma; CRC, Colorectal Cancer; DEGs, Differentially Expressed Genes; DT, Decision Tree; GBM, Gradient Boosting Machine; GNB, Gaussian Naive Bayes; GSE; HR, Hazard Ratio; KM, Kaplan–Meier; KNN, K-Nearest Neighbors; KIRC, Kidney Renal Clear Cell Carcinoma; LASSO, Least Absolute Shrinkage and Selection Operator; LDA, Linear Discriminant Analysis; lncRNAs, Long Non-Coding RNAs; LR, Logistic Regression; LUAD, Lung Adenocarcinoma; LUSC, Lung Squamous Cell Carcinoma; MCFS, Monte Carlo Feature Selection; MI, Mutual Information; mRMR, Minimum Redundancy Maximum Relevance; NB, Naive Bayes; PAAD, Pancreatic Adenocarcinoma; PCA, Principal Component Analysis; PPI, Protein–Protein Interaction; READ, Rectum Adenocarcinoma; RFE, Recursive Feature Elimination; RF, Random Forest; RSF, Random Survival Forest; SVM, Support Vector Machine.

In RNA-Seq datasets exhibiting imbalanced classes, ensemble models founded upon gradient boosting algorithms evince the highest performance. Algorithms such as XGBoost, CatBoost, and LightGBM have proven especially effective in classifying multiple tumor subtypes, including scenarios where rare tumour variants are represented by only a few samples [55,56]. The CatBoost algorithm has been shown to be particularly advantageous when working with categorical features, as demonstrated in a study predicting prostate cancer metastasis. A model based on RNA-Seq data from fresh-frozen tumor tissue samples achieved an AUC of 0.86 upon external validation using quantitative PCR on independent clinical formalin-fixed paraffin-embedded (FFPE) samples [13].

It is also worthy of note that specialized ensemble models, whose application in oncology research may be of particular interest, have been developed. In the work by Wallis et al., an ensemble classifier named M&M was developed, which classified 52 different types of pediatric tumors and 96 molecular subtypes with high accuracy and sensitivity, effectively encompassing both common and rare tumor subtypes [44]. The M&M algorithm synthesizes the outputs of two distinct models: a “Minority” model for the identification of rare tumors and a “Majority” model for the identification of common tumors. The synthesis process employs RF and k-nearest neighbours (kNN) algorithms. In the development of this algorithm, particular attention was paid to the process of feature selection. For the Minority classifier, the ANOVA method was applied, followed by the selection of the most significant transcripts based on F-statistic values. The RF algorithm was then used. In the Majority classifier, features were selected based on maximum transcript variance, followed by dimensionality reduction using PCA. The validation of the algorithm on both internal and external cohorts confirmed the reliability and versatility of this approach, demonstrating high rates of accuracy (up to 99%) and reproducibility (up to 77%).

**Figure 2:**
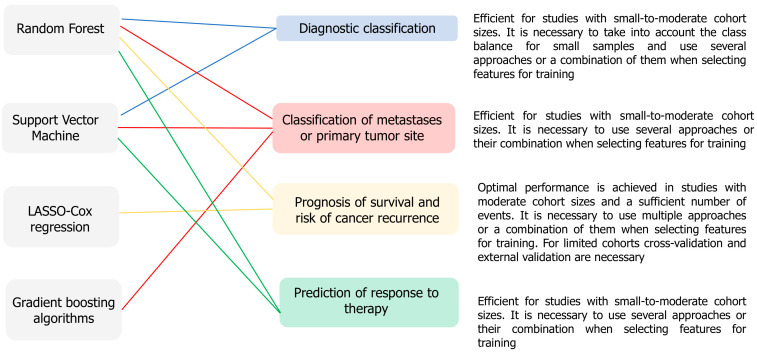
Associations of conventional ML algorithms with oncology research problems, according to the main trends in the analyzed studies. The diagram links frequently used algorithms to diagnostic classification, metastasis/primary site classification, prognosis of survival and recurrence risk, and prediction of response to therapy.

Based on the studies we reviewed on model development using traditional ML algorithms, we identified the integration of basic feature selection methods (LASSO or RF) with bioinformatics approaches such as pathway enrichment analysis or tumor microenvironment assessment using CIBERSORT as a strength. This allows models to achieve high accuracy rates and identify new significant biological mechanisms. However, critical limitations include the retrospective design of the studies with small sample sizes, which significantly increases the risk of model overfitting, despite the use of cross-validation. Furthermore, the lack of prospective clinical trials limits the implementation of these models in clinical practice. Overall, the studies reviewed highlight the relevance of using traditional ML algorithms to solve typical problems in oncology research, but require the inclusion of external validation and comparison with existing clinical tools (Figure 2).

## 4. Deep Learning Algorithms for Cancer Diagnostics, Prognostics and Classification

Distinct from conventional ML methodologies outlined above, which demand meticulous feature engineering and selection, DL algorithms automatically extract features from raw RNA-Seq data. DL-based models facilitate the analysis of substantial data volumes, the identification of intricate dependencies, and the automated processing of diverse omics data. At present, they are attracting increasing attention due to their flexibility and versatility, as they can be used for various research tasks, including classification, regression, and dimensionality reduction (Table 2). However, training DL models necessitates substantial data volumes; consequently, in scenarios where sample sizes are limited, conventional ML algorithms remain more prevalent [57].

Simple fully connected neural networks with multiple hidden layers have been shown to be capable of solving classification tasks for a variety of cancer types with a high degree of effectiveness. An example of such an architecture is the DeepCC model, where raw expression data are first transformed into functional spectra via biological pathway activity analysis, after which a neural network is applied to classify molecular subtypes [58]. A comparison of the performance of DeepCC with that of traditional classification methods on independent datasets comprising over 4000 samples was undertaken, the results of which demonstrated the former to be highly accurate. The model has been shown to achieve an accuracy rate in excess of 90% for established classification systems, including the CMS for colorectal cancer and the PAM50 for breast cancer [59,60]. Furthermore, the DeepCC model can be trained on these systems and subsequently used to classify new samples, ensuring reliability and reproducibility. A significant benefit of DeepCC, in contrast to other molecular tests such as MammaPrint, Oncotype DX, and BluePrint, is the generation of a biologically interpretable feature space, wherein patients are categorized according to the functional characteristics of their tumors [60,61,62].

As the number of layers increases, simple neural networks transform into deep neural networks (DNNs), thereby allowing such architectures to model more complex non-linear dependencies in RNA-Seq data. A particularly noteworthy example of such a structure is the DeSide model, which employs a five-layer architecture for the purpose of deconvoluting the cellular composition of solid tumors. DeSide has demonstrated the capacity to accurately identify 16 distinct cell types [63]. DNN has been demonstrated to be effective tools for predicting survival prognosis, especially when it integrates RNA-Seq data and clinical characteristics. For instance, in a study by Lai et al., a DNN model was developed to assess the survival of patients with non-small cell lung cancer. This model combines transcriptomic and clinical features through separate neural network branches, subsequently merging them in shared layers. The model achieved an AUC of 0.82, a figure that denotes a significant improvement in performance when compared with the RF algorithm on data sets of identical composition [64].

Worth mentioning are autoencoders, a special class of neural networks that can automatically identify key expression patterns, eliminating the need for labeled data. Autoencoders within hybrid architectures that combine various dimensionality reduction methods demonstrate high performance. In the PCA-AE-AdaBoost model, for instance, the authors first applied linear dimensionality reduction via principal component analysis, then non-linear reduction via an autoencoder, and finally classified the data using the AdaBoost ensemble classifier [65]. This multi-stage architecture demonstrated consistent performance across five independent breast cancer datasets, surpassing the performance of both standalone autoencoders and traditional machine learning methods. In turn, variational autoencoders incorporate a probabilistic interpretation into their architecture, thereby making the feature space more structured and interpretable. Vibert et al. presented the TransCUPtomics model, which uses a variational autoencoder to classify cancers of unknown primary [66]. Based on 94 different types of normal and tumor tissues, this model showed 96% accuracy and successfully classified 79% of these real cases.

Finally, the convolutional neural network (CNN) is the most widely used DL algorithm for solving various research tasks. Originally designed for image analysis, CNNs are now actively used for working with diverse genomic data thanks to various architectural modifications. A model based on a simple CNN architecture achieved an AUC of 0.99 for diagnosing breast cancer, with perfect specificity, outperforming the SVM algorithm when using the same data [67]. Another interesting approach to modifying the CNN algorithm is to use one-dimensional convolutions, which are more suitable for sequential data. In the TULIP model architecture, a 1D-CNN with two convolutional layers (each containing 128 filters) was used to classify 32 different tumor types [68]. Notably, this model achieved 97.6% accuracy when trained on just 19,758 protein-coding genes, outperforming a model that used 60,483 genes.

Bio-inspired optimization of CNNs via heuristic algorithms for working with bulk RNA-Seq data has also been demonstrated in the context of breast cancer. In a study by Mohamed et al., a diagnostic model based on a CNN algorithm with heuristic optimization (the Ebola Optimisation Search Algorithm) was developed which demonstrated high classification metric values and outperformed standard CNN algorithms. The authors note that the developed model more effectively identifies rare samples, reducing the number of false-negative classifications [69]. Another example of highly accurate diagnostic model development using RNA-Seq data is the study by Yaqoob et al., which proposed a hybrid feature selection approach combining heuristic algorithms that mimic the behavior of living systems—Harris Hawk Optimisation (HHO) and Whale Optimization Algorithm (WOA)—with a deep learning model [70]. The authors further optimized the selected features using a combined HHWO algorithm to eliminate irrelevant transcripts and enhance classification accuracy. The final model demonstrated 100% accuracy when using 2000 genes, and maintained high accuracy even when the number of features increased to 10,000. This indicates the model’s robustness to high data dimensionality.

Modern ML models for cancer classification are limited by the requirement for large annotated datasets, such as TCGA. However, replicating the scale of this project for new tumor types is impractical. Furthermore, there will never be a sufficient number of samples for training traditional models for rare cancers. To address this issue, Mostavi et al. proposed a one-shot learning strategy that uses minimal data to classify previously unknown tumor types. Within this framework, they developed the CancerSiamese model, which is based on Siamese Convolutional Neural Networks (SCNNs) and is trained on paired samples [71]. This model analyses the similarity between the transcriptomic profile of a query sample and reference tumors from known databases, using sample pairs for training. Rather than classifying samples into a fixed set of categories, CancerSiamese determines which known tumor type a new sample’s transcriptomic profile is most similar to. This approach significantly reduces the need for large annotated cohorts, which is particularly valuable when studying rare and under-researched tumors.

Despite the capabilities of DL models, several important nuances require careful consideration. First, despite the high performance of these models, they often reflect only internal validation results. In particular, studies demonstrating high performance metrics require careful interpretation. While such results can be achieved through careful feature selection and appropriate study design, it is still important to consider the biological complexity of cancer, limited sample sizes, and the typical challenges of RNA-seq. Given these points, the near-perfect metrics presented may reflect artifacts of internal validation or small-sample biases rather than the true generalization ability of the model. Therefore, when evaluating the results of the studies presented in Table 1 and Table 2, high AUC and accuracy values should be considered as indications of potentially promising development approaches that require independent external validation for confirmation. Moreover, when directly compared in similar study designs, deep learning models do not always outperform traditional machine learning methods. The RF algorithm demonstrates comparable performance on similar datasets, calling into question the need to use such complex architectures for typical research tasks. Furthermore, most deep learning models suffer from interpretability issues. For example, despite the high dimensionality reduction efficiency of autoencoder-based models, the biological meaning of hidden representations remains undiscovered without the inclusion of additional bioinformatics tools, such as for assessing significant biological pathways. In comparison, using LASSO/Elastic Net or RF algorithms with built-in feature importance estimation is clearly superior. Finally, deep learning algorithms are the most sensitive to data size. Typical sample sizes in the analyzed studies based on bulk RNA-Seq data average approximately 200–500 samples, primarily based on the TCGA database. Furthermore, sample sizes for rare tumor types often comprise fewer than 100 samples, and pan-cancer classifier models trained on numerous datasets may exhibit performance degradation on these samples. Deep learning algorithms require large datasets to avoid the risk of overfitting or significantly reduced accuracy on independent datasets, and traditional algorithms such as RF or SVM are generally preferred, in part due to their stability and reduced reliance on hyperparameters.

**Table 2:** RNA-Seq-based models developed using DL algorithms in the field of oncology encompasses the domains of diagnosis, prognosis and classification.

Model	Model Type	Datasets and Cohort Size	Sample Type	Model Algorithms	Metrics	Interpretability	Key Model Features	External Validation	Reference
DeepCC	Cancer subtyping, classification	CRC: TCGA-CRC train *n* = 456, 13 external CRC cohorts total *n* ≈ 3122; BRCA: TCGA-BRCA (*n* = 517) + multiple external microarray cohorts (*n* = 230).	Tumor tissues	Feedforward ANN with hidden layers; input: functional spectra from GSEA	CRC subtyping (TCGA-CRC): balanced accuracy > 90%; BRCA intrinsic subtype classification (TCGA-BRCA): balanced accuracy > 80%	Yes (deep features analyzed via Pearson correlation with GSEA scores; clustering identifies biological processes)	Platform-independent; transforms expression to functional spectra for batch effect robustness; single sample prediction with adaptive rescaling; learns hierarchical features	Yes (13 independent CRC datasets; 4 breast cancer datasets)	[58]
DeSide	Cellular deconvolution	TCGA tumors samples (*n* = 7699); merged 12 scRNA-seq datasets rom tumor tissues and cancer cell lines (*n* = 325,474 single cells)	Tumor tissue and cancer cell lines	DNN with two 7-layer MLPs	DeSide-derived cell-type scores significantly stratify overall survival in multiple TCGA cohorts (Cox *p* < 0.005).	Yes (integration of biological pathways for feature extraction)	Unified model for multiple solid tumors; predicts non-cancer cells first; handles intra/inter-tumor heterogeneity	Yes (SC_HNSC, SC_GBM, SC_OV, GSE184398)	[63]
PCA-AE-Ada	Prognosis	GSE2034 train/validation *n* = 266, external: GSE4922 *n* = 133, GSE6532 *n* = 100, GSE7390 *n* = 190, GSE11121 *n* = 182	Tumor tissues	PCA + stacked autoencoder; concatenated features to AdaBoost	10 × 5-fold CV on GSE2034 and independent tests on four GEO cohorts: ACC 0.72–0.85, AUC 0.68–0.74 (mean AUC ≈ 0.71), sensitivity 0.68–0.84, specificity 0.55–0.66	No	Combines linear (PCA) + non-linear (autoencoder) feature learning with ensemble boosting; handles high-dimensional, noisy, heterogeneous data; alleviates class imbalance; data alignment via zero-padding	Yes (4 independent GEO datasets: GSE11121, GSE1456, GSE4922, GSE6532)	[65]
TransCUPtomics	Diagnostics, classification	Reference: 20,918 RNA-seq samples (39 cancer types, 55 normal tissues) from TCGA, GTEx, Human Protein Atlas, GSE60052, GSE118014; CUP cohort: 48 patients (37 retrospective, 11 prospective)	Fresh-frozen tumor tissue	VAE + RF + k-NN	Reference set: overall accuracy ≈ 96% for tissue-of-origin prediction (three-fold cross-validation); CUP cohort: tissue of origin predicted in 79% (38/48) cases; in the prospective cohort, 7/8 (87.5%) patients treated according to TransCUPtomics showed objective responses	Yes (VAE latent features via gene weights and GO analysis)	Integrates TOO with gene fusion and variant detection; confidence scoring; handles normal cell contamination; UMAP visualization	Yes (37 retrospective + 11 prospective CUP patients)	[66]
Lightweight CNN	Classification	TCGA-BRCA (*n* = 1208)	Tumor and normal tissues	Lightweight CNN with 2 convolutional layers, max pooling, batch normalization, dense layers	5-fold CV on TCGA-BRCA: Accuracy 98.8%, Sensitivity 91.4%, Specificity 100%, Precision 100%, F1 = 0.955, AUC = 0.998	No	Transforms gene expression → 2D images edge detection for visualization; lightweight for small samples and high dimensions; GC normalization, gene filtering	No	[67]
TULIP	Classification (primary tumor type prediction)	TCGA (32 types, *n* = 10,940)	Tumor tissue	1D-CNN with 2 conv layers, max pooling, 2 FC layers, dropout 10%	Internal train/validation/test splits: overall test accuracy 94.7–97.6%; weighted precision/recall/F1 ≥ 0.92; accuracy > 90% for 16/17 and ≥80% for 28–29/32 tumor types; external CPTAC kidney cohort: all 277 samples classified as kidney cancer	No	Python QC tool (version 3.7.12) for unknown tumor prediction	Yes (CPTAC kidney cancer)	[68]
EOSA-CNN	Classification	TCGA-BRCA (*n* = 1208)	Tumor and normal tissues	Hybrid: CNN optimized by Ebola Optimization Search Algorithm (EOSA)	Balanced accuracy = 0.959, overall accuracy = 98.3%, precision ≈ 0.90, recall/sensitivity ≈ 0.93, F1 ≈ 0.91, Cohen’s kappa ≈ 0.90; class-wise sensitivity and F1 for tumor class ≈ 99%	No	Bio-inspired optimization for high dimensionality and imbalance; outperforms standalone CNN, GA-CNN, WOA-CNN	No	[69]
HHWO-DL	Classification	RNA-Seq from 66 paired samples from 55 patients + 11 controls	Paired tumor and normal tissues	Hybrid gene selection: mRMR + HHWO optimization; multi-layer feedforward NN	Mean accuracy ≈ 99%, ROC AUC ≈ 0.99	No	Hybrid optimization for gene selection reduces dimensionality and avoids local optima	No	[70]
CancerSiamese	Classification	TCGA primary tumors (29 types; *n* = 10,340 samples) and MET500 metastatic cohort (20 types; *n* = 765 samples); training on 19 primary and 10 metastatic cancer types, testing on disjoint unseen types	Tumor tissues	Siamese CNN with parallel 1D-CNNs joined by similarity metric network; trained on paired samples	Unseen primary tumors: one-shot 6-/8-/10-way accuracy 89.67%, 87.32%, 84.59%; metastatic tumors: ≈63–66% accuracy	Yes (Guided Backpropagation Saliency Maps for gene significance; stepwise greedy forward selection for markers (top 100 primary, 200 metastatic); DAVID for GO/KEGG/Biocarta pathways)	One-shot learning for primary and metastatic tumor types unseen in training; network transfer learning from pretrained 1D-CNN	Yes (unseen cancer types disjoint from training; cross-application)	[71]

Abbreviations: ANN, artificial neural network; AUC, area under the curve; BRCA, breast cancer; CNN, convolutional neural network; CRC, colorectal cancer; CUP, cancer of unknown primary; DNN, deep neural network; GO, Gene Ontology; GSEA, gene set enrichment analysis; HHO, Harris Hawk Optimization; HHWO, Hybrid Harris Hawk and Whale Optimization; HR, hazard ratio; k-NN, k-nearest neighbors; mRMR, minimum redundancy maximum relevance; NB, Naive Bayes; OS, overall survival; PCA, principal component analysis; RF, random forest; SVM, support vector machine; TOO, tissue of origin; VAE, variational autoencoder; WOA, Whale Optimization Algorithm.

## 5. Current Trends in the Development of Cancer ML Models Based on Transcriptomic Data

As previously discussed, various ML model architectures based on bulk RNA-Seq data demonstrate high performance when it comes to addressing current challenges in cancer research. However, these models are limited by the nature of bulk RNA-Seq data itself: signals are averaged across diverse cellular components within a tumor sample. Consequently, valuable information about rare cell populations is lost [72].

In this section, we focus on recent trends that extend beyond bulk RNA-Seq–only models and are organized into three thematic directions: (1) ML and DL models based on scRNA-Seq data, (2) integrative approaches combining bulk and single-cell transcriptomic profiles, and (3) recent developments in multi-omics ML models.

Single-cell RNA sequencing technology opens up new possibilities for analyzing tumor transcriptomes, enabling the analysis of gene expression profiles of individual cells and revealing cellular heterogeneity that is inaccessible with bulk RNA-seq [73,74]. Specialized databases are being developed to handle this data type. Among these, scCancerExplorer is a leading resource, integrating standardized scRNA-Seq data from various cancer types and offering tools for interactive analysis [75]. Other public resources include CancerSEA, TISCH2 and the Single Cell Portal [36,76].

In the context of developing advanced ML models, scRNA-Seq data allows for the incorporation of tumor cellular heterogeneity. Algorithms based on graph neural networks (GNNs) have been successfully applied to cell clustering, the identification of rare subpopulations and the reconstruction of tumor cell differentiation trajectories [77,78,79]. For example, the scGNN model is highly effective at clustering and gene expression imputation, including the analysis of cell types in cancer [80]. The Gossip Flow model was developed to diagnose primary liver tumors by clustering and analyzing intercellular communication [81].

Using transformer algorithms to analyze scRNA-Seq data facilitates the development of pre-trained models that can transfer knowledge and estimate tumor heterogeneity at the single-cell level. The scGREAT model, for instance, is a pre-trained deep language model based on a transformer that can infer gene regulatory networks from scRNA-Seq data. Gene embeddings in this model effectively model regulatory interactions, enabling the identification of key pathways in tumor cells [82]. Another transformer-based model, scFoundation, was pre-trained on over 50 million gene expression profiles [83]. This model demonstrates high performance in predicting responses to anti-cancer drugs and in classifying tumors at the single-cell level.

As most existing databases contain predominantly bulk RNA-Seq data, methods for integrating bulk and scRNA-Seq data are becoming increasingly important in research. One example of combining these transcriptomic data types is the T-GEM transformer model, which is adapted to predict phenotypes based on gene expression while accounting for the unordered nature of the genes and their interactions [84]. A similar model is PERCEPTION, which was developed to predict responses to targeted anti-cancer therapies [85].

Although this review primarily focuses on the use of bulk RNA-Seq data in ML model development, it is important to acknowledge the growing trend of utilizing multi-omics data in model construction [86,87]. Notable developments in oncology include models created for cancer diagnosis, prognosis and survival prediction (e.g., Pathformer [88]; PathCNN [89]) and biomedical classification (MOGONET [90]), all of which are based on integrative multi-omics data analysis. The Pathformer model can be additionally used for tumor diagnosis via liquid biopsy.

Despite scRNA-Seq and multi-omics data representing cutting-edge areas of oncology research, they remain costly and less accessible. For most research groups and clinical laboratories, bulk RNA-Seq data is more readily available. In this context, computational deconvolution methods for bulk RNA-Seq data are a critical alternative for extracting information about tumor cellular composition from existing datasets [91]. These methods open up possibilities for oncology research, such as assessing immune cell infiltration in tumor tissues, determining the tumor-to-stromal component ratio, and identifying prognostically significant cellular subpopulations.

Among deconvolution algorithms that estimate cell-specific gene expression directly from bulk RNA-Seq data without the need for scRNA-Seq, CIBERSORTx is a notable tool. It uses support vector regression-based machine learning to quantify 22 immune cell types [92]. The DSSC tool uses a sample similarity matrix to improve deconvolution accuracy, particularly for rare cell types [93]. BayesPrism was specifically developed for oncology applications, employing Bayesian integration to account for tumor-specific expression changes [94]. Reference-free deconvolution methods such as TOAST and DeClust also merit mention [95,96]. Such approaches are particularly important when studying rare tumors for which single-cell reference atlases are unavailable.

Integrating deconvolution with machine learning methods yields significant results. The Kassandra model, which is based on a decision tree algorithm for cellular deconvolution, can virtually reconstruct the cellular composition of tissues from RNA-Seq data [84]. This model can identify over 50 unique cell types, including complex T-cell subpopulations. Furthermore, by accounting for technical noise and aberrant expression in cancer cells and employing quantitative normalization to transform RNA levels into absolute cell numbers, Kassandra demonstrates high prediction accuracy and applicability to various biopsy types. It can also predict responses to immunotherapy based on the tumor microenvironment, highlighting its potential for clinical diagnostics.

## 6. Practical Recommendations for Developing Cancer ML Models Based on Bulk RNA-Seq Data

**Figure 3:**
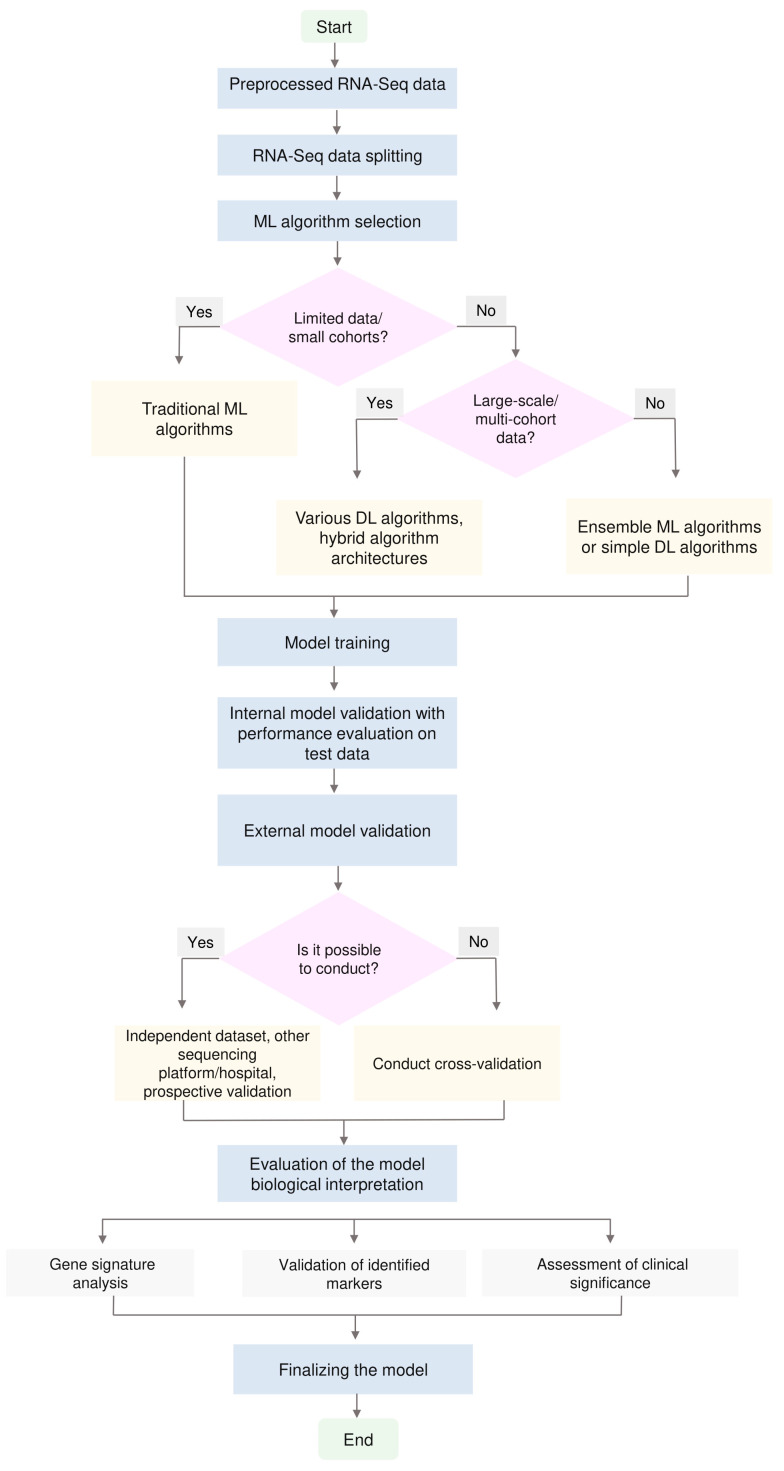
A schematic representation of the RNA-Seq ML pipeline for cancer research. Starting from preprocessed RNA-Seq data, the dataset is split into training and test sets, followed by the selection of an appropriate ML algorithm. The blocks “Limited data/small cohorts?” and “Large-scale/multi-cohort data?” emphasize that model complexity should be chosen in accordance with the amount and diversity of the available data. In settings with limited data, simpler, well-regularized traditional algorithms are preferable, whereas as the size and heterogeneity of the datasets increase, it becomes reasonable to employ ensemble methods, simple DL architectures or more hybrid DL approaches. After algorithm selection, model training and internal validation with performance assessment on the test set are performed, and external validation is conducted whenever possible. The final stages include biological interpretation of the model and its finalization for potential translational application.

Developing effective ML models based on bulk RNA-Seq data requires a systematic approach that considers sample size, task type and available computational resources. Based on a critical analysis of the literature, we present practical recommendations for developing such models (Figure 3).

First of all, it is important to emphasize that when choosing ML algorithms, it is not so much the absolute sample size that is important, but rather the “effective information volume,” which is determined by the number of observations and outcomes, the degree of class imbalance, the signal-to-noise ratio, the dimensionality of the feature space, the presence of batch effects, the inherent complexity of biological relationships, and the planned complexity of the model [97]. Modern methodological studies show that simple empirical rules do not take these factors into account and do not ensure the stability and transferability of predictive models [97,98]. With limited data, when the number of informative observations is small relative to the dimensionality of the feature space, regularized models of moderate complexity are preferable—penalized regression (LASSO/Elastic Net), RF, SVM—in combination with careful feature selection for compact gene panels. These algorithms generally demonstrate higher resistance to overfitting and lower variability of estimates in small samples. As data volume and heterogeneity increase, the use of more flexible ensemble methods (XGBoost, LightGBM) and simple DNN architectures becomes justified, especially in cases of pronounced class imbalance or in multi-class classification problems. In such cases, ensemble approaches such as M&M and pathway-oriented neural networks such as DeepCC can be useful, increasing the biological interpretability of results [43,56].

Large multi-cohort datasets make it possible to apply more complex DL architectures capable of automatically extracting multi-layer representations and integrating heterogeneous data [99]. The choice of architecture is determined by the research task: autoencoders are used for unsupervised learning and dimensionality reduction, CNNs for analyzing structured representations, and hybrid models for integrating heterogeneous sources. However, even with large samples, DL models remain sensitive to overfitting, require careful hyperparameter tuning and internal validation, and generally have lower interpretability than traditional methods [100,101]. To assess the sufficiency of the available data volume, it is advisable to rely on formalized approaches to calculating sample size for the development and validation of predictive models. These approaches take into account model complexity, outcome frequency, and acceptable levels of overfitting, and can also utilize learning curves and information-theoretic criteria [97,98]. Such methods allow one to assess whether the existing cohort ensures the reliable development and reproducible external validation of clinically oriented models.

The next crucial aspect is selecting an appropriate strategy for each research task. For example, for binary classification tasks, the most optimal solution for achieving high accuracy and interpretability would be the use of a combination of the RF algorithm with LASSO feature selection. When developing diagnostic models, it is essential to evaluate performance metrics correctly. When the data is imbalanced with respect to the trait under study, precision and recall metrics must be used in addition to accuracy. For diagnostic models, recall is of the utmost importance. The F1-score balances precision and recall; a high F1-score indicates that the model detects most positive cases with high precision [102]. Another common metric in model development is the AUC-ROC, but this is also sensitive to imbalanced and small datasets. Using this metric in combination with accuracy, precision and recall allows for a better assessment of the clinical significance of the developed model [102].

When assessing the predictive performance of models, it is important to consider the potential instability of individual metrics, such as AUC-ROC. High metric values may only partially reflect the variability of small samples or dependence on the preprocessing pipeline used, rather than a robust biological signal. Various methodological studies recommend assessing model stability using approaches such as bootstrapping, repeated cross-validation, and feature stability analysis [103,104,105,106]. These approaches allow us to identify models whose performance changes significantly with minor modifications to the data or data analysis approaches. It is therefore advisable to report not only point estimates of performance metrics, but also simple measures of their variability, for example, confidence intervals or the spread of metric values obtained by bootstrap resampling. This approach provides a more complete understanding of the reliability of results and helps identify consistent patterns, thereby increasing their reproducibility and potential clinical applicability.

Classifying multiple cancer types or molecular subtypes is a more complex task, especially in the presence of rare variants and limited sample sizes. In such cases, gradient boosting algorithms or specialized ensemble approaches may be most effective. For tasks involving the prediction of patient survival, Cox regression with elastic net regularization can be used for small and medium-sized samples. Alternatively, for large samples, DNN architectures that integrate clinical parameters can be used. Models that combine transcriptomic data with clinical characteristics through separate neural network branches demonstrate substantially improved performance. It is important to account for censored survival data and use the appropriate loss functions.

When it is not possible to obtain experimental scRNA-Seq data, computational deconvolution of bulk RNA-Seq data can be used as an alternative method to assess the cellular composition of tumor samples. Moreover, the choice of deconvolution method depends on the availability of reference profiles; thus, in the presence of an scRNA-Seq atlas for the corresponding tumor type, the use of the CIBERSORTx or BayesPrism algorithms is recommended. If reference data is unavailable, reference-free methods such as TOAST or DeClust should be used instead. We also wish to note the fact that, for the identification of prognostically significant cellular subpopulations, the deconvolution results can be integrated with clinical data.

Reproducibility of results is a central aspect of model development, determining its clinical applicability and generalizability. However, the presence of various technical artifacts, methodological errors, and insufficient validation can significantly reduce its reliability. Researchers must primarily assess the presence of batch effects in the bulk RNA-Seq data they use, which can distort the data and significantly impact model performance. This is particularly relevant when incorporating multiple data sources, such as using data from TCGA and GEO databases to form a combined sample for training. A recommended step at this stage is the visual assessment of data using dimensionality reduction methods to identify sample groups based on technical characteristics, for example, through principal component analysis (PCA). If batch effects are present in the data, specialized tools must be employed to eliminate them.

The next critical issue is the use of information in the test dataset that was utilized in the training dataset. This phenomenon is known as data leakage and can lead to significant model overfitting and distortion of prediction results. To prevent data leakage, it is essential to adhere to the proper temporal sequence in data processing, such that data splitting should occur immediately after basic filtering of low-expressed genes, but before normalization, feature selection, and model training. All stages of RNA-Seq data preprocessing must be performed separately for the training sample, and then the same parameters applied to the test sample.

Finally, conducting external validation on independent cohorts is the gold standard for assessing the model’s generalizability and is critically important for transitioning from research developments to clinical application. Models that demonstrate high performance on internal cross-validation but exhibit a substantial decline in metrics on external validation indicate issues with overfitting or the influence of batch effects. Obtaining independent cohorts for validation can often be challenging, and in such cases, it is recommended to use public datasets with comparable study designs and clinical characteristics, for example, from TCGA and GEO databases.

In conclusion, we would like to note that high-quality development of models based on RNA-Seq data should transparently reflect the description of all data preprocessing stages, including the indication of the moment of splitting into training and test samples, demonstration of reproducibility through provision of code and description of model parameters, as well as external validation on an independent cohort. Adherence to all these conditions is critically important for the potential clinical implementation of the developed model. It is also worth noting that to increase confidence in the developed model, particularly from clinicians, it is recommended to conduct biological validation of identified markers. This may include functional enrichment analysis (GSEA) for identified gene signatures to confirm the involvement of relevant biological pathways, and finally, validation of key markers using qRT-PCR on an independent patient cohort.

## 7. Challenges and Future Prospects

Despite significant progress in developing RNA-seq-based ML models, there are fundamental limitations to their implementation in clinical practice. Technical variability and the quality of the source data are critical factors in determining the success of the models. The quality of RNA-seq data depends directly on the condition of the initial RNA samples, the sequencing depth and the technical preparation of the libraries [107]. Differences in RNA extraction protocols, library preparation methods, and sequencing platforms can lead to systematic errors. This problem is particularly acute when working with archival FFPE samples, where RNA degradation and chemical modifications can significantly impact the analyzed transcriptome profiles.

Reproducibility of model results across independent studies remains a central challenge. Models trained on one dataset often demonstrate reduced efficacy when applied to others. Data from TCGA is often inadequate for other datasets, such as those from GEO or the International Cancer Genome Consortium (ICGC), due to differences in protocols, sequencing platforms, population heterogeneity and biological context [108]. The development of universal, standardized pipelines that are robust to batch effects and cross-platform differences is currently of paramount importance. Methods for removing variability, correcting batch effects, and employing adaptive strategies can enhance the reproducibility and transferability of models between laboratories and medical institutions [12,109].

Furthermore, the high dimensionality of RNA-Seq data, coupled with small sample sizes, creates the so-called ‘curse of dimensionality’, which generates a high risk of model overfitting and the identification of false markers [99,110]. This problem is particularly acute for rare tumors and novel molecular subtypes. Approaches such as semi-supervised learning, one-shot learning and transfer learning offer a promising way forward when working with limited sample sizes. For example, the CancerSiamese model demonstrated the ability to classify rare tumors based on a small number of samples. However, such approaches require further validation for clinical application.

Finally, the limited interpretability of DL models represents a significant barrier to their implementation in clinical practice. While conventional ML algorithms, such as RF, provide direct feature importance estimates, complex neural network architectures function as opaque decision-making systems [111]. For successful integration into clinical practice, it is essential to ensure algorithm transparency and the ability to explain decisions. Therefore, enhancing model interpretability is one of the most important areas for development in this field [12,73,112]. This involves advancing interpretable machine learning (iML) approaches, including local explanation methods such as SHAP and LIME, as well as intrinsically interpretable models. Another promising approach is to develop architectures based on biological knowledge, such as knowledge-primed neural networks, where the connections between layers are defined by molecular. This enables the mapping of the model’s latent features to known biological processes. Furthermore, integrating iML approaches with the preliminary binarization of RNA-seq data demonstrates significant potential: this method maintains accuracy while greatly simplifying the interpretation of results, particularly when analyzing data across various cancer types [15,112,113].

## 8. Conclusions

The integration of transcriptome data with ML methods holds enormous potential for new advances in the development of diagnostic, prognostic, and therapeutic tools for various cancer types. The continued development of RNA-seq-based models will be determined by their ability to overcome existing technical and methodological limitations for clinical application. The pursuit of standardized RNA-seq data preparation protocols, the development of computational methods, the implementation of clinical validation, and close collaboration between researchers and clinicians will help realize the full potential of RNA-seq data and artificial intelligence for the qualitative development of personalized medicine. Unlike previous reviews, which primarily focus on DL models, this review emphasizes the specifics of using the most accessible and widely used bulk RNA-Seq data to create models, evaluating a variety of ML approaches. We highlight practical recommendations for developing models in oncology research and methods for deconvolving bulk RNA-Seq data as a promising alternative to scRNA-Seq, which remains inaccessible to a wide range of researchers. This review serves as a comprehensive guide for developing ML models from RNA-Seq data for clinical oncology.

## Data Availability

No new data were created or analyzed in this study. Data Availability Statement is not applicable.
